# Association between lipid-lowering drug targets and the risk of cystic kidney disease: a drug-target Mendelian randomization analysis

**DOI:** 10.1080/0886022X.2025.2491657

**Published:** 2025-04-27

**Authors:** Zhiwen Lian, Zijie Liang, Qiyan Chen, Chao Xie, Yaozhong Kong

**Affiliations:** Division of Nephrology, The First People’s Hospital of Foshan, Foshan, Guangdong, China

**Keywords:** Lipid-lowering drugs, statins, cystic kidney disease, mendelian randomization, genome-wide association study

## Abstract

**Background:**

Evidence regarding the causal relationship between lipid-lowering drugs and cystic kidney disease, including polycystic kidney disease (PKD), was limited. This study aimed to evaluate the causal relationship between lipid phenotypes mediated by lipid-lowering drug targets—3-hydroxy-3-methyl glutaryl coenzyme A reductase (HMGCR), proprotein convertase subtilisin/kexin type-9 (PCSK9), and Niemann-Pick C1-like 1 (NPC1L1)—and the risk of cystic kidney disease and PKD.

**Methods:**

Genetic variants encoding lipid-lowering drug targets—HMGCR, PCSK9, and NPC1L1—from published genome-wide association study (GWAS) statistics were collected to perform drug target Mendelian randomization (MR) analysis. Summary statistics for the GWAS of cystic kidney disease and PKD were obtained from the FinnGen consortium and the European Bioinformatics Institute. Inverse variance weighting (IVW) was used as the primary MR analysis method, with sensitivity analyses conducted to ensure the robustness of the results.

**Results:**

Increased gene expression of HMGCR was associated with an elevated risk of cystic kidney disease (IVW-MR: odds ratio [OR] = 3.05, 95% confidence interval [CI] = 1.19–7.84, *p* = 0.02) and PKD (IVW-MR: OR = 2.13, 95% CI = 1.01–4.46; *p* = 0.045). There was no evidence of causal effects of PCSK9 and NPC1L1 targets on cystic kidney disease and PKD. Cochran’s Q test, MR-PRESSO, and MR-Egger intercept tests showed no heterogeneity or horizontal pleiotropy among the instrumental variables.

**Conclusions:**

This study supported that increased HMGCR expression was associated with an increased risk of cystic kidney disease and PKD, suggesting potential benefits of statin therapy for cystic kidney disease and PKD. Further research is necessary to elucidate specific mechanisms and potential therapeutic applications of HMGCR inhibitors.

## Background

Cystic kidney disease encompasses a diverse array of hereditary and acquired conditions marked by unilateral or bilateral renal cysts [1]. Simple cysts, which occur singly or in small numbers, affect nearly 50% of individuals over 40 years of age [1]. In contrast, polycystic kidney disease (PKD) represents a clinically significant group of genetically driven disorders featuring prominent, enlarging, typically bilateral renal cysts [2]. PKD is categorized based on their inheritance patterns as dominant, recessive, or X-linked [3]. Large cysts are a defining feature of autosomal dominant polycystic kidney disease (ADPKD), the most prevalent form of PKD. In contrast, other types of PKD typically exhibit smaller cysts [4]. ADPKD, with a prevalence ranging from 1 in 400 to 1 in 2000, is the most common monogenic disorder in humans and contributes to approximately 8-10% of end-stage renal disease (ESRD) cases in the United States [5,6]. Given the high incidence and severe consequences of chronic kidney disease (CKD), it’s crucial to implement early detection and tailored treatment strategies to mitigate its major impact on renal health [7].

Lipid-lowering drugs have been widely used for the prevention and management of cardiovascular diseases. While statin therapy [3-hydroxy-3-methyl glutaryl coenzyme A reductase (HMGCR) inhibitors] is primarily used for cardiovascular protection based on its anti-atherosclerotic and endothelial protection benefits [8–10], its pleiotropic effects on cystic kidney disease warrant exploration. A systematic review and meta-analysis of five clinical studies revealed that statins did not significantly alter the progression of ADPKD in terms of estimated glomerular filtration rate (eGFR) and total kidney volume, however, they provided benefits in reducing urinary protein and serum low-density lipoprotein levels [11]. A randomized, placebo-controlled trial is currently underway to evaluate the efficacy of pravastatin in slowing kidney disease progression in adult patients with early-stage ADPKD [12], while a study in normal volunteers and those with mild-to-moderate ADPKD showed no significant changes in renal blood flow after four weeks of simvastatin therapy [13].

Potential mechanisms linking lipid-lowering drug targets with cystic kidney disease have revealed several intriguing pathways that may represent novel therapeutic avenues. Recent research shows that HMGCR inhibition protects endothelial cells by suppressing YAP-mediated chromatin remodeling, which is crucial for preventing endothelial-to-mesenchymal transition [14]. Additionally, HMGCR inhibition extends beyond cholesterol reduction, encompassing anti-inflammatory and antioxidant properties that are particularly beneficial in the context of cystic kidney disease [15,16]. HMGCR inhibition may modulate signaling pathways, such as the mTOR and Wnt/β-catenin pathways, which are known to be hyperactive in cystic kidney diseases, thereby potentially influencing cyst formation and progression [17–20]. The role of proprotein convertase subtilisin/kexin type-9 (PCSK9) in renal disease is an area that has garnered increasing attention. Previous research has demonstrated that elevated PCSK9 levels exacerbate renal inflammation in mouse models exposed to high-fat diets and streptozotocin, as well as in cultured renal epithelial cells [21]. PCSK9 was also implicated in diabetic nephropathy by inducing mitochondrial DNA damage and activating the cGAS/STING pathway, which amplifies inflammatory responses [22]. These findings suggest that PCSK9 inhibitors may have a protective role in renal diseases characterized by inflammation and oxidative stress, including cystic kidney disease. Indeed, the role of lipid-lowering agents, particularly PCSK9, and Niemann-Pick C1-like 1 (NPC1L1) inhibitors, in cystic kidney disease remains understudied. Based on the potential mechanisms, we hypothesize that specific lipid-lowering drug could interact with these drug targets, potentially exerting its effects in cystic kidney disease.

Mendelian randomization (MR) leverages the principle of random genetic allocation at conception. This approach mimics randomized controlled trials (RCTs) by reducing confounding bias and reverse causality inherent in observational studies [23]. With the rapid advancement of basic theory and the proliferation of applications, drug-target MR analysis is gradually becoming an efficient tool for inferring the effect of agents targeting protein-encoding genes [24,25]. By employing these genetic variants as proxies for exposure, we can estimate the potential effect of drug targets on disease risk. Therefore, the aim of this study was to evaluate three lipid-lowering drug targets—HMGCR, PCSK9, and NPC1L1—was associated with a decreased risk of cystic kidney disease and PKD.

## Materials and methods

### Study overview and data source

HMGCR, PCSK9, and NPC1L1 are all involved in lipid metabolism and have been investigated as potential drug targets for decreasing low-density lipoprotein cholesterol (LDL-C) [26]. HMGCR is the target of statins, PCSK9 the target of alirocumab and evolocumab, and NPC1L1 is target of ezetimibe (a drug used to lower cholesterol absorption). The LDL-C genetic dataset was selected from the Global Lipids Genetics Consortium, the largest lipid genome-wide association study (GWAS) meta-analysis comprising approximately 173,082 individuals of European ancestry [27,28]. The outcome data for cystic kidney disease was obtained from the FinnGen consortium (website: https://www.finngen.fi/en), which includes 469 cases of cystic kidney disease patients and 217,979 controls [29]. Cystic kidney disease is defined as a congenital or acquired kidney disorder characterized by the presence of renal cysts. The genetic dataset of PKD was obtained from the European Bioinformatics Institute (website: https://www.ebi.ac.uk/gwas/downloads/summary-statistics), comprising 424 cases of PKD patients and 355, 431 controls [30]. Information on the database and ICD-10 code for defining on cystic kidney disease were described in Table 1 and Tables S1 and S2. The study followed the Strengthening the Reporting of Observational Studies in Epidemiology Using Mendelian Randomization (STROBE-MR) guidelines [31]. Ethical approvals and informed consent were secured for the original GWAS studies. We utilized publicly available genetic data at the summary level, obviating the need for additional ethical approvals.

**Table 1.** The data source used for MR analysis in this study.

Phenotype	Case	Control	Year	Ancestry	Sources
Exposure					
Genetic proxies for lipid- lowering drugs in Europeans	173,082	individuals	2021	European	GLGC Consortium
Outcome					
Cystic kidney disease	469	217, 979	2021	European	FinnGen GWAS
Polycystic kidney disease	424	355, 431	2021	European	EBI GWAS

Abbreviations: GLGC, Global Lipid Genetics Consortium; GWAS, Genome-wide association study; EBI, European Bioinformatics Institute.

**Table 2.** Heterogeneity and pleiotropy test in the examined associations and weak instrument statistics between lipid phenotypes mediated by lipid-lowering drug targets (HMGCR, PCSK9, and NPC1L1) and cystic kidney disease and polycystic kidney disease.

Exposure	Cochran’s Q			Rucker’s Q			MR-Egger intercept			MR-PRESSO
	Q	df	P-value	Q	df	P-value	Intercept	se	P-value	P-value (Global test)
Cystic kidney disease										
LDL-C	351.41	388	0.91	352.25	389	0.91	0.01	0.01	0.36	
HMGCR	2.36	5	0.80	2.36	6	0.88	−0.01	0.15	0.94	0.91
PCSK9	7.05	10	0.72	7.26	11	0.78	−0.02	0.03	0.66	0.78
NPC1L1	0.68	1	0.41	2.11	2	0.35	0.38	0.31	0.44	/
Polycystic kidney disease										
LDL-C	408.14	389	0.24	409.14	390	0.24	−0.01	0.01	0.33	
HMGCR	5.79	5	0.33	5.88	6	0.44	−0.04	0.13	0.79	0.58
PCSK9	5.28	10	0.87	6.73	11	0.82	0.04	0.04	0.26	0.80
NPC1L1	1.13	1	0.29	1.98	2	0.37	0.30	0.35	0.55	/

Notes: Cochran’s Q with a one-side P-value of < 0.05 were considered as an indication of heterogeneity. A significant difference (one-side *p* < 0.05) between the Cochran’s Q and Rucker’s Q (Q-Q’) was considered to indicate the MR-Egger test to be a better method to study the genetic association between the particular exposure and outcome. MR-PRESSO test and an MR-Egger’s intercept of zero, tested using a single side P-value threshold of >0.05, was considered to provide evidence for absence of pleiotropic bias.

P of Global test was not available for the analysis of NPC1L1 due to not enough instrumental variables.

Abbreviations**:** SE, standard errors; MR-PRESSO, Mendelian randomization pleiotropy residual sum and outlier.

### Selection of genetic instruments for proxying lipid-lowering drug targets

**Figure 1.**
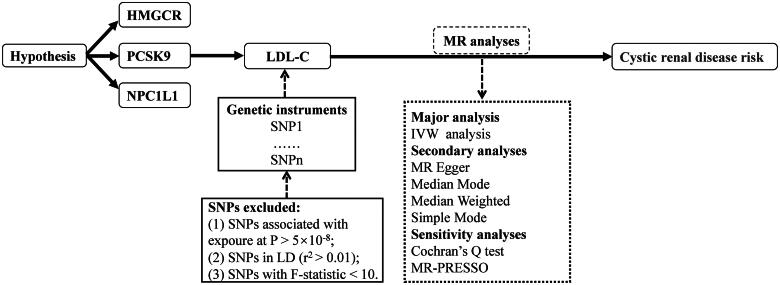
Flowchart of the current MR design.

The selection of LDL-C-associated genetic instruments was based on criteria including selecting single nucleotide polymorphisms (SNPs) associated with LDL-C by setting a significance threshold (*p* < 5.0 × 10^−8^) and minor allele frequency (MAF) > 1%. Additionally, a linkage disequilibrium (LD) clumping (r^2^ < 0.01 and a maximum physical distance of 10,000 kilobases) was identified to minimize the probability of genetically linked SNPs. The genes encoding the pharmacological targets of lipid-lowering drugs were screened in the DrugBank database (website: https://go.drugbank.com/). Specifically, we identified genetic variants within ± 100 kb distance around each target gene locus in lipid traits that were associated with LDL-C at genome-wide significance (*p* < 5 × 10^−8^). To avoid the influence of strong LD on the results, a threshold for LD was set (r^2^ < 0.3). The HMGCR is assembly in GRCh37.p13: chromosome 5:74632993-74657941 (URLs: https://www.ncbi.nlm.nih.gov/gene/3156), the PCSK9 is assembly in GRCh37.p13: chromosome 1: 55505221-555305252 (URLs: https://www.ncbi.nlm.nih.gov/gene/255738), and the NPC1L1 is assembly in GRCh37.p13: chromosome 7: 44552134-44580929 (URLs: https://www.ncbi.nlm.nih.gov/gene/29881). To assess potential weak instrumental bias for lipid-lowering drug targets, F statistics were calculated using the following formula: F = R^2^/(1 - R^2^) * (N-K-1)/K, where R^2^ is the cumulative explained variance of the SNPs in the exposure database, N represents the sample size, and K is the number of SNPs. SNPs with F-statistics < 10 were excluded, as an F-statistic of at least 10 is considered the minimum threshold to mitigate weak instrumental bias [32]. Finally, we conducted the MR analysis on the genetically instrumented lipid-lowering drug targets, evaluating their causal effects on cystic kidney disease and PKD risk. Figure 1 shows the hypothesis and process of the MR study.

### Sensitivity analysis

Sensitivity analysis was used to assess potential heterogeneity and pleiotropy. Cochran’s Q and Rucker’s Q test were performed to evaluate the heterogeneity of SNPs. A Cochran’s Q and Rucker’s Q test *P*-value < 0.05 indicates significant heterogeneity in the analysis. MR-Egger regression intercept was used to assess the presence of horizontal pleiotropy. Horizontal pleiotropy refers to the potential influence of instrumental variables on the outcome through biological pathways unrelated to the exposure factor. The *P*-value < 0.05 in the MR-Egger regression indicated significant horizontal pleiotropy in the analysis. In addition, MR-PRESSO (Mendelian Randomization Pleiotropy RESidual Sum and Outlier) was employed to identify horizontal pleiotropy. In cases of pleiotropy detection, an outlier adjustment was applied to refine the effect estimates. To assess the robustness of our MR analysis, we employed the ‘leave-one-out’ method, where each genetic instrument was sequentially removed, and the MR analysis was repeated. Additionally, the funnel plots were conducted to visually inspect the potential for small study effects or publication bias, which can also indicate the presence of outlier instruments.

### Statistical analysis

In primary MR analysis to assess the causal effects of the target genes on cystic kidney disease and PKD, we performed the Wald Ratio method for those with a single IV and the inverse variance weighted (IVW) method based on the fixed-effect model for those with two or more IVs. If heterogeneity across SNPs exists, a random-effect model was used in the IVW method. IVW method integrates the Wald ratio causal estimates derived from individual SNPs and generates an aggregated causal impact of the exposure on the outcome through a meta-analytic framework [33,34]. In contrast to the conventional fixed-effects IVW, the random-effects IVW method offers a more conservative causal inference by incorporating uncertainties arising from pleiotropy. To further ensure the robustness of our findings, we then compared the pattern of IVW results with other MR methods. These additional analyses were performed to verify the consistency of our results with the primary analysis. We conducted the statistical power calculations on MR analyses using an online tool (https://shiny.cnsgenomics.com/mRnd/). A sufficient power of over 80% was recommended. All data analyses were conducted in R software (version 4.3.0, R Foundation for Statistical Computing, Vienna, Austria) using the TwoSampleMR package (version 0.5.7). The study results were presented as odds ratio (OR) with 95% confidence intervals (CI), with a significance level of *p* < 0.05.

## Results

### Selection of genetic instruments

After removing SNPs in linkage disequilibrium with LDL-C, a total of 390 SNPs related to LDL-C were included as genetic instruments. Seven SNPs were selected as proxies for the HMGCR gene, with F-statistics ranging from 47.6 to 156.9. Twelve SNPs were selected as proxies for the PCSK9 gene, with F-statistics ranging from 44.2 to 152.4. Three SNPs were included for genetically proxied NPC1L1 gene, with F-statistics ranging from 52.1 to 80.8. Across the drug-target instruments examined, the F-statistics were > 10 and the variance explained by the instruments ranged from 0.13% to 0.75%, indicating that weak instrument bias was unlikely to affect the analyses. Additional details on the harmonized SNPs are provided in the Supplementary data.

### Association of genetic proxies for lipid-lowering drug targets and on cystic kidney disease

**Figure 2.**
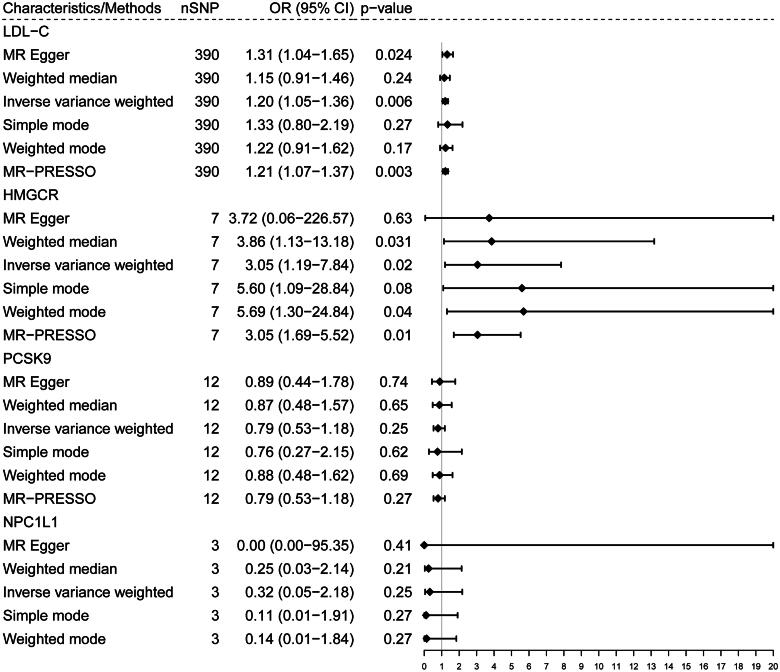
Forrest plot for associations of genetically predicted genes targets of lipid-lowering drugs with risk of cystic kidney disease. The causal effect estimation of NPC1L1 targets using MR − PRESSO was not available because of the impact of limited number of IVs and variance explained. No potential outlier was detected by the MR-PRESSO (outlier-corrected) test.

The IVW method showed that genetically predicted LDL-C levels were significantly associated with an increased risk of cystic kidney disease (IVW-MR: OR = 1.20, 95% CI = 1.05–1.36, *p* = 0.006; Figure 2). Increased gene expression of HMGCR was associated with an elevated risk of cystic kidney disease (IVW-MR: OR = 3.05, 95% CI = 1.19–7.84; *p* = 0.02; Figure 2). In addition, MR analyses using weighted median, weighted mode, and MR-PRESSO further showed similar findings and confirmed the robustness of these results (all *p* < 0.05; Figure 2). MR-Egger analysis did not show a causal association between HMGCR and cystic kidney disease (*p* > 0.05), but the results were directionally consistent with the other four MR methods (OR > 1; Figure 2). The statistical power was calculated as >80%, indicating a low risk of weak instrument bias in the MR analyses. Scatter plots also showed that HMGCR was closely aligned with the regression line for cystic kidney disease, as shown in Figure S1. However, none of the genetic proxies for lipid-lowering drugs (PCSK9 and NPC1L1) targets were found to be causally associated with the risk of cystic kidney disease (Figure 2 and Figure S1).

### Association of genetic proxies for lipid-lowering drug targets and on PKD

**Figure 3.**
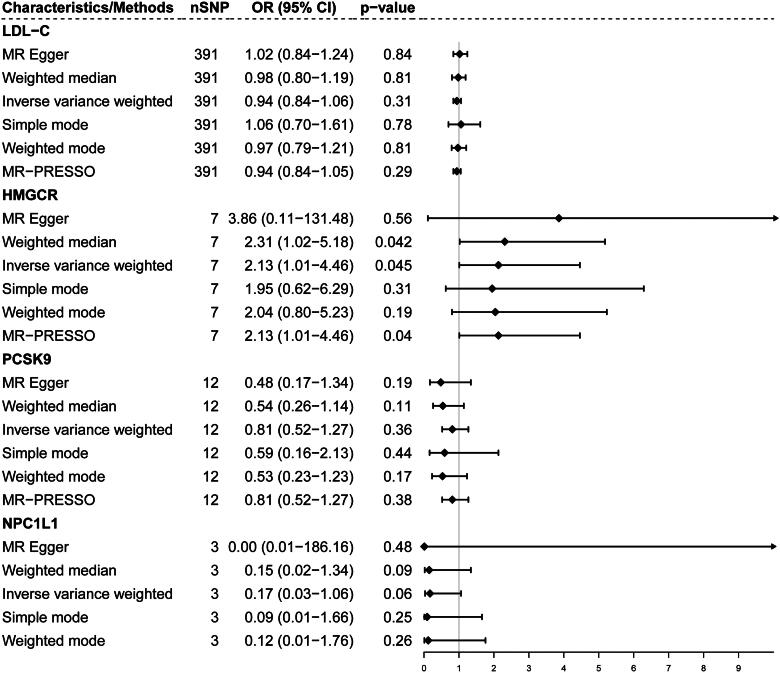
Forrest plot for associations of genetically predicted genes targets of lipid-lowering drugs with risk of polycystic kidney disease. The causal effect estimation of NPC1L1 targets using MR − PRESSO was not available because of the impact of limited number of IVs and variance explained. No potential outlier was detected by the MR-PRESSO (outlier-corrected) test.

The primary analysis and weighted median showed that increased gene expression of HMGCR was associated with an increased risk of PKD (IVW-MR: OR = 2.13, 95% CI = 1.01–4.46; *p* = 0.045; weighted median: OR = 2.31, 95% CI = 1.02–5.18; *p* = 0.042; Figure 3). Although the secondary analyses—simple mode, weighted mode, MR-Egger analysis, and MR-PRESSO—did not show statistical differences, these analyses were directionally consistent. Notably, we did not find any other genetic proxies for PCSK9 and NPC1L1to be causally associated with PKD (Figure 3 and Figure S2).

### Sensitivity analysis

As shown in Table 1, no significant heterogeneity was observed by Cochran’s Q heterogeneity test. MR-Egger intercept analysis did not detect any potential horizontal pleiotropy (HMGCR, *p* = 0.85; PCSK9: *p* = 0.73; NPC1L1: *p* = 0.93). No outlier SNPs were detected using MR- PRESSO methods, and no horizontal pleiotropy or heterogeneity was observed in the analysis results. Forest plots showed the MR effect sizes of HMGCR-, PCSK9-, and NPC1L1-mediated LDL-C levels on cystic kidney disease and PKD (Figures S3 and S4). The leave-one-out analysis showed consistent results across all iterations (Figures S5 and S6), indicating that no single genetic instrument significantly influenced the overall findings. This suggests that the observed associations between lipid-lowering drug targets and the risk of cystic kidney disease are robust and not unduly influenced by any single genetic instrument. Funnel plots did not show any evidence of asymmetry, further supporting the absence of publication bias and the robustness of our results (Figures S7 and –S8).

## Discussion

The present study investigated the association between lipid-lowering drug targets and cystic kidney disease and PKD using MR analysis focused on drug targets. We showed that increased genetically proxies for HMGCR were associated with elevated risk of cystic kidney disease and PKD. However, we found no evidence of causal effects regrading increased gene expression of PCSK9 and NPC1L1 targets and cystic kidney disease and PKD.

Cystic kidney disease comprises a diverse array of genetic and acquired conditions characterized by the presence of cystic lesions in the kidneys, which exhibit varying properties and pathophysiological features [2]. These disorders encompass inherited, developmental, and acquired etiologies [3]. Metabolomic studies have shown marked differences in lipid metabolism between patients with PKD and healthy individuals [35]. Current lipid-lowering drugs often focus on HMGCR, NPC1L1, and PCSK9 as common targets [26]. These drugs modulate the activity or expression of molecules to varying degrees to achieve lipid-lowering goals [36]. As HMG-CoA reductase inhibitors, statins reduce cholesterol levels and also have various additional effects, such as influencing immune responses [37,38]. These include altering the infiltration of white blood cells into tissues, modulating the presentation of antigens and inhibiting T-cell activation [37,38].

Previous studies have shown both a single high loading dose of atorvastatin administered 24 h before contrast media exposure and high-dose rosuvastatin demonstrate significant protective effects against contrast-induced acute kidney injury (CI-AKI), with atorvastatin reducing CI-AKI in patients at low to medium risk and rosuvastatin showing enhanced benefits in ACS patients with higher baseline hs-CRP levels [39,40]. Additionally, a recent multicenter cohort study indicated that in patients with type 2 diabetes mellitus, statin initiation is associated with reduced risks of developing diabetic kidney disease and kidney function decline, particularly when high-intensity LDL-C control is achieved [41]. In the context of cystic kidney disease, particularly ADPKD, existing literature suggests that statins do not significantly impact disease progression in terms of eGFR and total kidney volume. However, there is evidence indicating that statins may offer benefits by reducing urinary protein levels and serum LDL-C levels [11]. Ongoing research, including RCTs such as those evaluating pravastatin, aims to further assess the efficacy of statins in slowing kidney disease progression in adult ADPKD patients with early-stage disease [12,13]. A phase III RCT has evaluated the efficacy of pravastatin in slowing ADPKD progression in children and young adults. The trial measured changes in height-corrected total kidney volume, left ventricular mass index, and urine microalbumin excretion over three years and found that pravastatin significantly reduces the progression of kidney cyst growth compared to placebo [42]. Additionally, a *post hoc* analysis has examined the safety of concurrent use of tolvaptan and statins in ADPKD patients, finding no significant increase in statin-related adverse events or hepatic transaminase elevations associated with tolvaptan compared to placebo-treated subjects [43]. Despite these findings, research on the potential of statin therapy to prevent cystic kidney disease remains limited. The current study suggests that among various classes of lipid-lowering agents, only statins demonstrate potential in reducing the risk of cystic kidney disease.

The pathogenesis of cystic kidney disease and PKD involves a multifaceted interplay of genetic mutations, aberrant cell signaling, epithelial cell dysfunction, inflammation, lipid metabolism alterations, hemodynamic changes, and epigenetic modifications. The precise mechanism by which HMGCR inhibition is associated with a reduced risk of cystic kidney disease remains elusive. Based on the current study results, several hypotheses can be postulated. Firstly, oxidized LDL-C and lipid abnormalities are central to kidney damage, promoting inflammation, oxidative stress, cellular senescence and endothelial dysfunction [15,16,44]. By reducing LDL cholesterol and improving lipid balance, statins mitigate lipid-induced oxidative stress and inflammation, thereby preserving kidney health and potentially slowing disease progression [15,16]. Secondly, recent study also showed that statins inhibit endothelial-to-mesenchymal transition in heart and kidney microvascular endothelial cells by altering chromatin accessibility and enhancing endothelial function through epigenetic pathways. These findings highlight statins’ broader therapeutic potential in conditions linked to endothelial dysfunction [14]. Thirdly, statins protect podocytes, the specialized cells maintaining the filtration barrier, by inhibiting apoptosis, supporting cellular survival, and stabilizing the cytoskeleton [45]. This preservation of podocyte function is critical for minimizing kidney damage and treating conditions such as CKD. These effects are expected to affect signal transduction, cell growth, and cell polarity [46]. Collectively, these mechanisms suggest that statins offer multifaceted kidney protection, potentially improving outcomes in patients with kidney cysts who are at risk for renal impairment [36].

To note, the present study of genetically proxied inhibitors of NPC1L1 and PCSK9 failed to show a protective effect against the risk of cystic kidney disease. These findings highlight the multiple mechanisms underlying the targets of lipid-lowering drugs in the pathogenesis of cystic kidney disease. The protease PCSK9 plays a critical role in the regulation of cholesterol homeostasis, primarily by interacting with and degrading LDL receptors [47]. Preclinical studies indicate that elevated PCSK9 levels following podocyte injury contribute to lipid abnormalities in nephrotic syndrome, while PCSK9 deficiency ameliorates these changes [48,49]. Additionally, PCSK9 expression in the kidney exacerbates hypercholesterolemia in nephrotic syndrome and impairs megalin-mediated protein reabsorption in CKD, thereby aggravating proteinuria [50]. These findings suggest that PCSK9 inhibitors may offer therapeutic potential for hypercholesterolemia and proteinuria in nephrotic syndrome and CKD. Despite significant reductions in LDL-C with PCSK9 inhibitors, there is clinical trials on their effects on kidney diseases. Despite PCSK9 inhibitors effectively lowering LDL-C, our findings suggest that targeting PCSK9 alone may not sufficiently mitigate cystic kidney disease progression, possibly due to its limited role in renal-specific pathways. NPC1L1, a cholesterol uptake transporter expressed in the small intestine and liver, is selectively inhibited by NPC1L1 inhibitors at the brush border of the small intestinal mucosa. This reduces cholesterol absorption from food and bile, thereby lowering plasma and liver cholesterol levels [51]. These targets might have a weaker direct effect on kidney physiology compared to HMGCR. NPC1L1 primarily functions in lipid metabolism, particularly in LDL-C regulation, but their roles in renal pathophysiology might be less direct or pronounced.

To note, our study based on data from European individuals, may limit the generalizability of our findings to other ethnic groups due to population heterogeneity. Genetic variants used as instruments can have different allele frequencies and effect sizes in non-European populations, potentially affecting the strength and validity of our genetic instruments. Additionally, the prevalence and management of lipid disorders, as well as the response to lipid-lowering drugs, can vary across ethnic groups. Similarly, the prevalence and genetic etiology of cystic kidney disease differ across populations, which may influence the observed associations. Future studies in diverse ethnic groups are essential to confirm our findings and understand the broader implications of lipid-lowering drug targets on cystic kidney disease risk.

Our study also has several limitations. First, the use of genetic variation to study the effects of lipid-lowering drugs is limited by the lifelong and modest effects of these drugs, whereas pharmacological intervention tends to have more pronounced effects for an individual. Therefore, the MR analysis does not reflect the short-term effects of taking lipid-lowering drugs. Secondly, while the proportion of variance explained is relatively low, this is typical for MR analyses involving complex traits. The F-statistic for the HMGCR instrument was well above the threshold of 10, indicating that it is sufficiently strong to avoid weak instrument bias. Thirdly, although Cochran’s Q test and MR-Egger regression indicated no significant heterogeneity or horizontal pleiotropy, latent pleiotropy arising from undetected gene-environment interactions or other unmeasured confounders remains a potential limitation. This could introduce bias in the observed associations between lipid-lowering drug targets and the risk of cystic kidney disease. Fourthly, the small sample size of NPC1L1 genetic instruments may introduce biases such as weak instrument bias. Even sensitivity analyses were conducted to reveal significant heterogeneity or asymmetry, the small sample size remains a critical consideration. Finally, our study focused exclusively on two major classes of cystic kidney diseases and PKD due to their high prevalence and clinical significance, acknowledging the potential limitations of generalizing the findings to other cystic renal conditions with distinct etiologies.

## Conclusions

In conclusion, our MR analysis showed the beneficial influence of focusing on HMGCR inhibition and cystic kidney disease and PKD risk. Conversely, PCSK9 and NPC1L1 did not show a clear protective benefit. Further studies are essential to elucidate the precise mechanisms and potential therapeutic implications of statins in cystic kidney disease prevention.

## Supplementary Material

Supplement file R4.doc

## Data Availability

The datasets analyzed for this study are available on the website (https://gwas.mrcieu.ac.uk and https://www.finngen.fi/fi). All data accessed and analyzed in this study are publicly available and included in supplementary materials.
