# Supplementary material for: Association between lipid-lowering drug targets and the risk of cystic kidney disease: a drug-target Mendelian randomization analysis
Source: Ren Fail. 2025 Apr 27;47(1):2491657. doi: 10.1080/0886022X.2025.2491657 (PMC12035922; doi:10.1080/0886022X.2025.2491657)
Supplement: Supplement file R4.doc [file IRNF_A_2491657_SM4313.doc]

**Association between lipid-lowering drug targets and the risk of cystic kidney disease: A drug-target Mendelian randomization analysis**

|  |  |
| --- | --- |
| Supplemental Figure S1 | Scatter plot of genetic association of lipid- lowering drug targets and cystic kidney diseases risk using five MR methods. |
| Supplemental Figure S2 | Scatter plot of genetic association of lipid- lowering drug targets and PKD risk using five MR methods. |
| Supplemental Figure S3 | Forest plot of genetically casual association of lipid-lowering drug targets with cystic kidney diseases. |
| Supplemental Figure S4 | Forest plot of genetically casual association of lipid- lowering drug targets with PKD. |
| Supplemental Figure S5 | Leave-One-Out analyses for variants associated with lipid- lowering drug targets on cystic kidney diseases. |
| Supplemental Figure S6 | Leave-One-Out analyses for variants associated with lipid- lowering drug targets on PKD. |
| Supplemental Figure S7 | Funnel plot for the effects of lipid-lowering drug targets on cystic kidney diseases. |
| Supplemental Figure S8 | Funnel plot for the effects of lipid-lowering drug targets on PKD. |
| Supplemental Table S1 | Basic information on lipid-lowering drugs. |
| Supplemental Table S2 | ICD-10 code for cystic kidney disease (Q17_CYSTIC_KIDNEY_DISEA) |

| 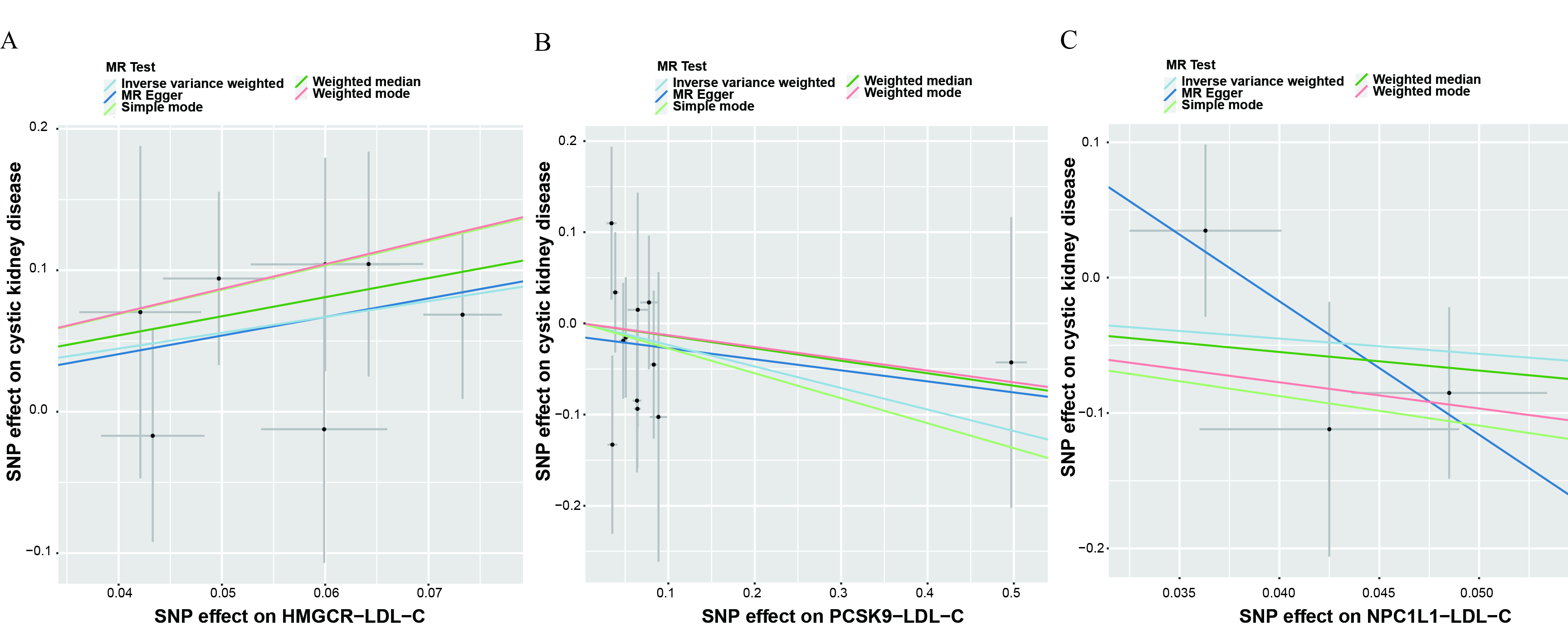  Supplementary Figure S1. Scatter plot of genetic association of lipid-lowering drug targets and cystic kidney diseases risk using five MR methods.  Notes: Scatter plot including the MR estimates between lipid-lowering drug targets and cystic kidney diseases, in which a threshold of P < 1 × 10^−8^ was used for the selection of variants. The variants’ effect on lipid-lowering drug targets were displayed on X-axis, the variants effect on cystic kidney diseases were displayed on the Y-axis. The gradient of each line represents the MR estimate for the corresponding model and the error bars represent standard errors of effect sizes. (A) Scatter plot of SNPs effects on HMGCR−LDL−C versus their effects on cystic kidney diseases. (B) Scatter plot of SNPs effects on PCSK9−LDL−C versus their effects on cystic kidney diseases. (C) Scatter plot of SNPs effects on NPC1L1−LDL−C versus their effects on cystic kidney diseases. |
| --- |


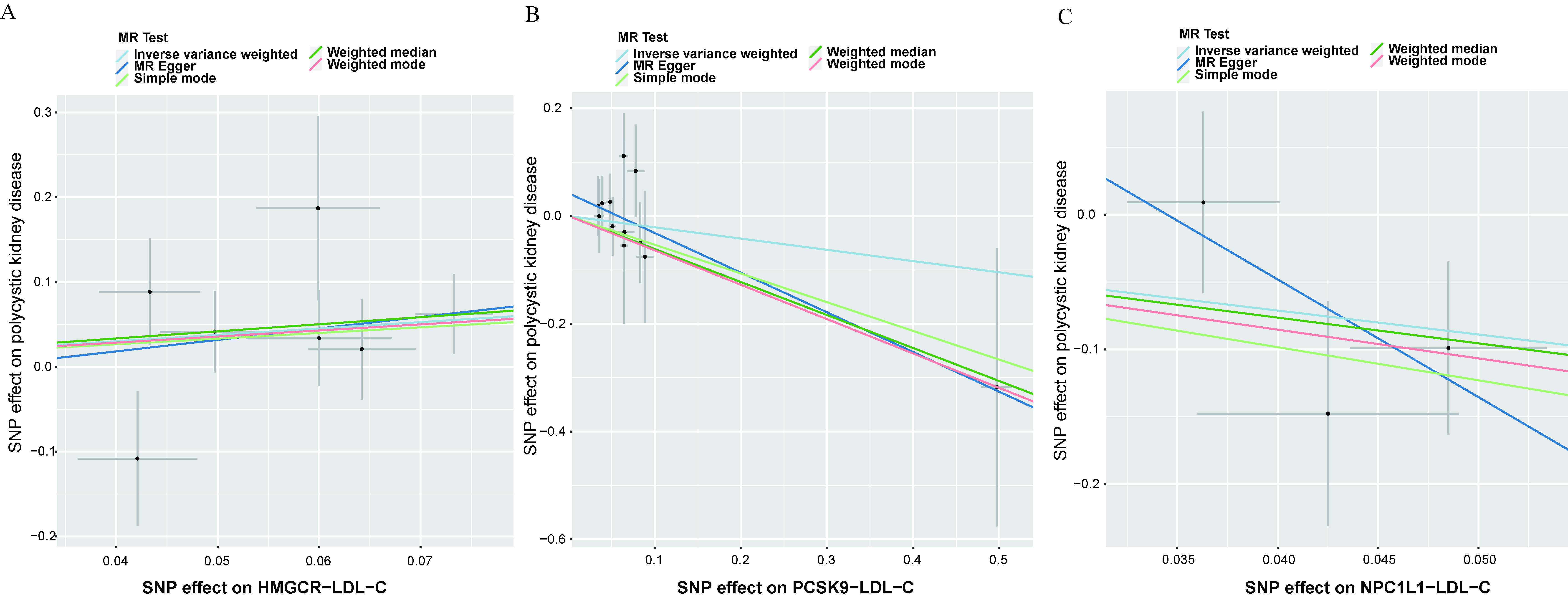


Supplementary Figure S2. Scatter plot of genetic association of lipid-lowering drug targets and PKD risk using five MR methods.

Notes: Scatter plot including the MR estimates between lipid-lowering drug targets and PKD, in which a threshold of P < 1 × 10^−8^ was used for the selection of variants. The variants’ effect on lipid-lowering drug targets were displayed on X-axis, the variants effect on PKD were displayed on the Y-axis. The gradient of each line represents the MR estimate for the corresponding model and the error bars represent standard errors of effect sizes. (A) Scatter plot of SNPs effects on HMGCR−LDL−C versus their effects on PKD. (B) Scatter plot of SNPs effects on PCSK9−LDL−C versus their effects on PKD. (C) Scatter plot of SNPs effects on NPC1L1−LDL−C versus their effects on PKD.


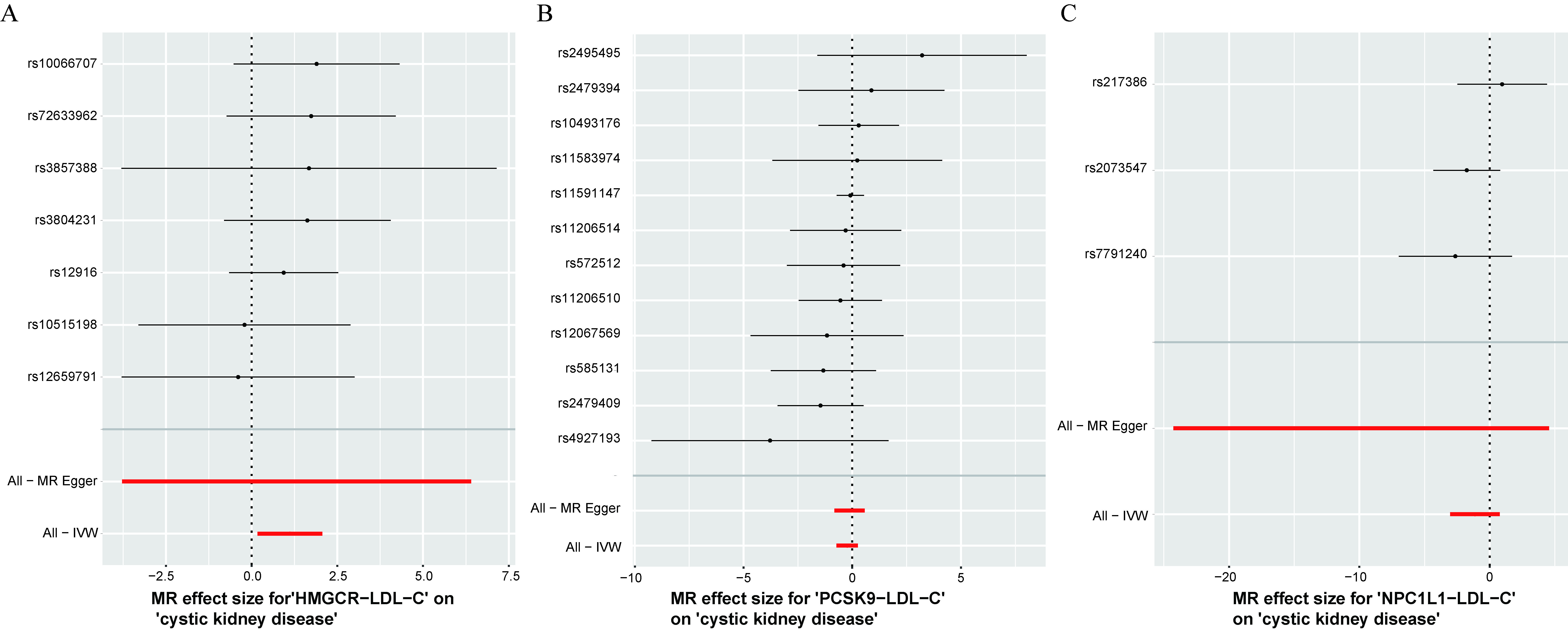


Supplementary Figure S3. Forest plot of genetically casual association of lipid- lowering drug targets with cystic kidney diseases.

Notes: The MR effect size of lipid-lowering drug targets were displayed on the X-axis. The different genetic variants for cystic kidney diseases were listed on the Y-axis. The red point showed the casual effect estimate of lipid-lowering drug targets and cystic kidney diseases with valid SNPs utilizing the Egger or inverse variance weighted method, and the red lines indicated the 95% CI of the estimate. (A) Forest plot of genetically casual association of HMGCR−LDL−C with cystic kidney diseases. (B) Forest plot of genetically casual association of PCSK9−LDL−C with cystic kidney diseases. (C) Forest plot of genetically casual association of NPC1L1−LDL−C with cystic kidney diseases.


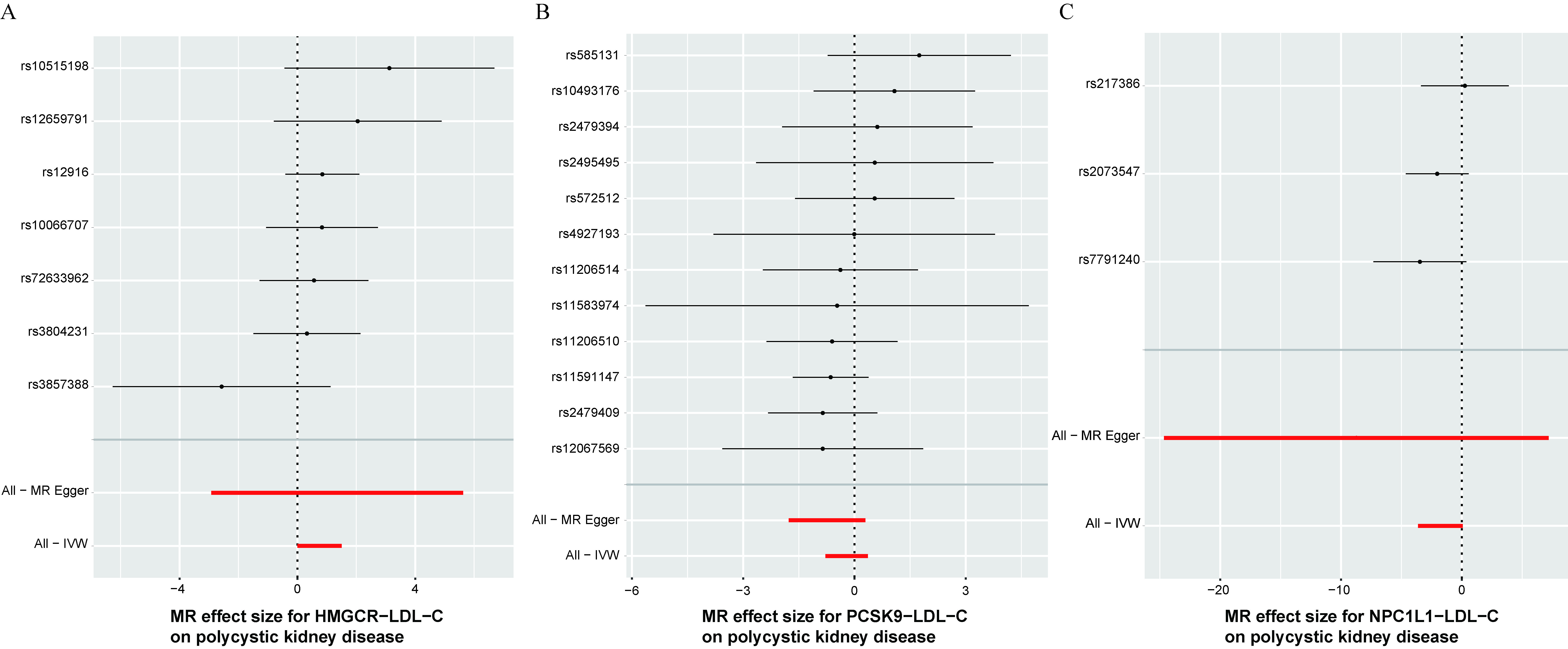


Supplementary Figure S4. Forest plot of genetically casual association of lipid-lowering drug targets with PKD.

Notes: The MR effect size of lipid-lowering drug targets were displayed on the X-axis. The different genetic variants for PKD were listed on the Y-axis. The red point showed the casual effect estimate of lipid-lowering drug targets and PKD with valid SNPs utilizing the Egger or inverse variance weighted method, and the red lines indicated the 95% CI of the estimate. (A) Forest plot of genetically casual association of HMGCR−LDL−C with PKD. (B) Forest plot of genetically casual association of PCSK9−LDL−C with PKD. (C) Forest plot of genetically casual association of NPC1L1−LDL−C with PKD.


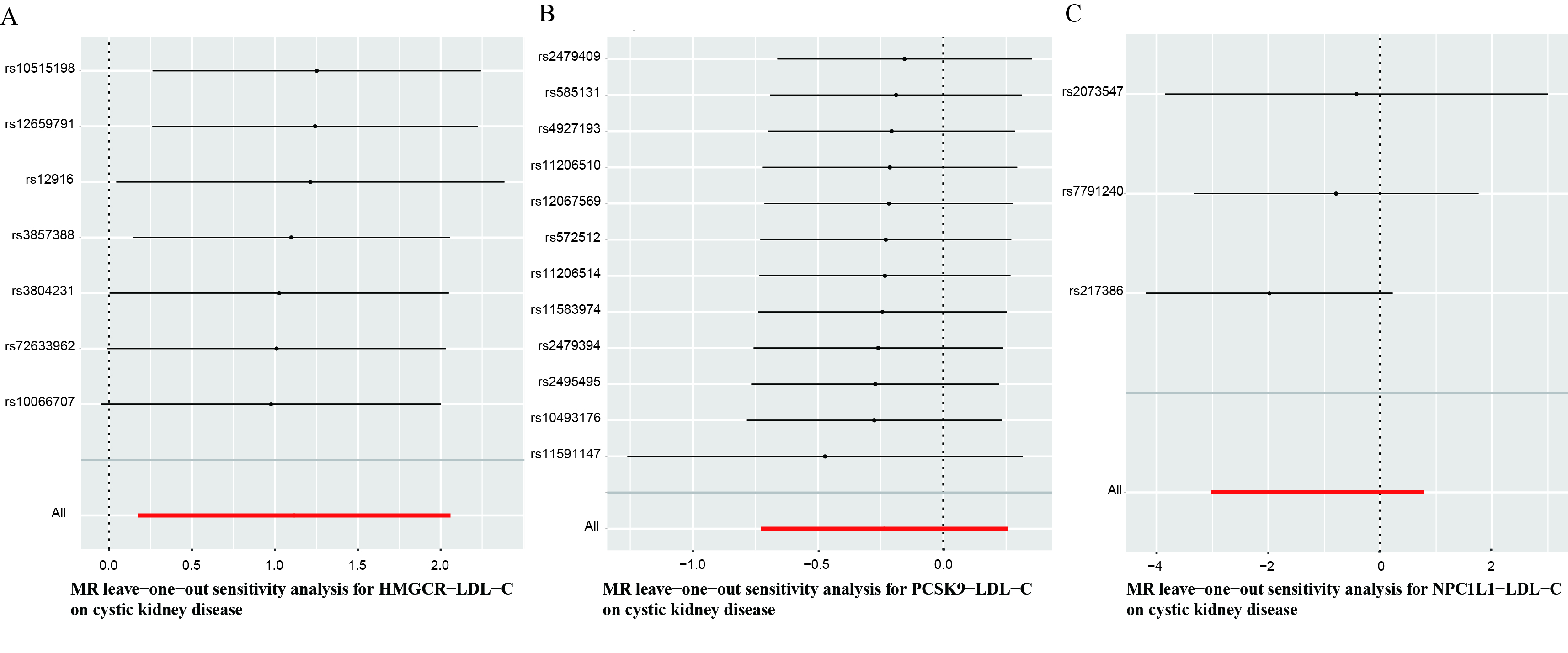


Supplementary Figure S5. Leave-One-Out analyses for variants associated with lipid-lowering drug targets on cystic kidney diseases.

Notes: The influence of single variant on the overall MR analysis of cystic kidney diseases was indicated by sequential removal of each genetic variant respectively in HMGCR−LDL−C (A), PCSK9−LDL−C with PKD (B), NPC1L1−LDL−C (C).


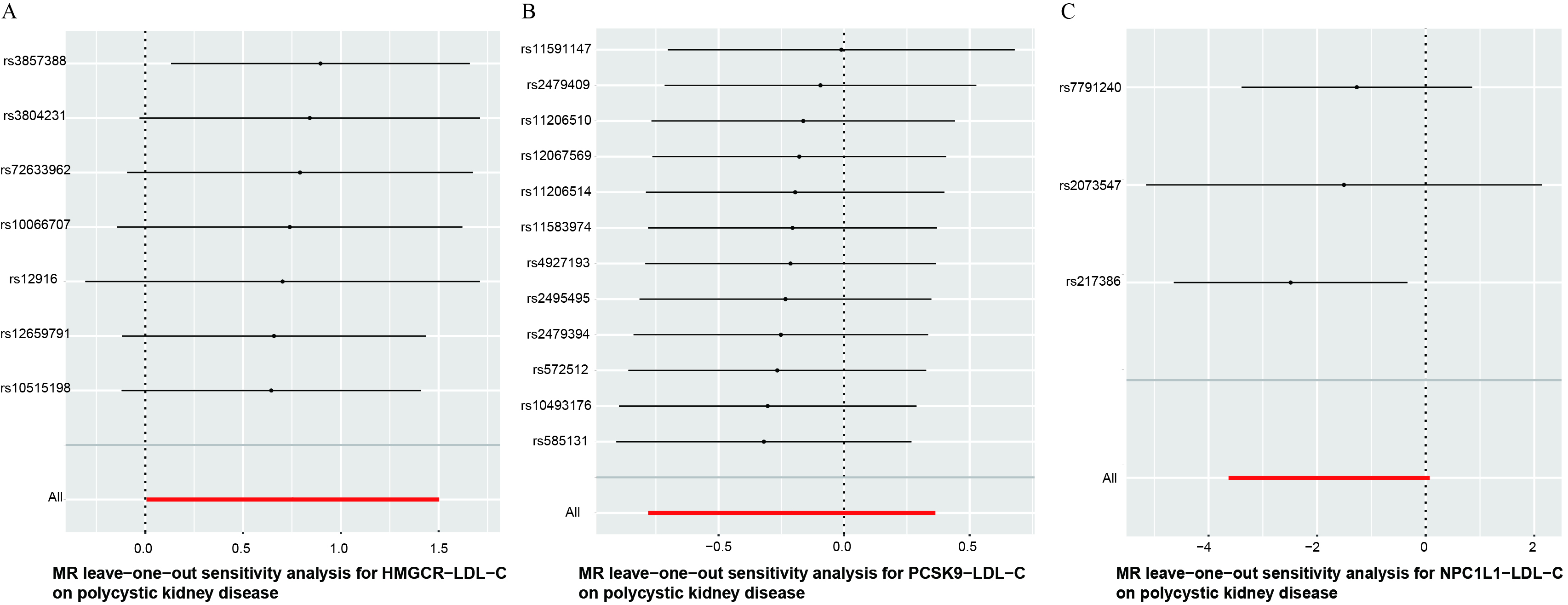


Supplementary Figure S6. Leave-One-Out analyses for variants associated with lipid-lowering drug targets on PKD.

Notes: The influence of single variant on the overall MR analysis of PKD was indicated by sequential removal of each genetic variant respectively in HMGCR−LDL−C (A), PCSK9−LDL−C with PKD (B), NPC1L1−LDL−C (C).


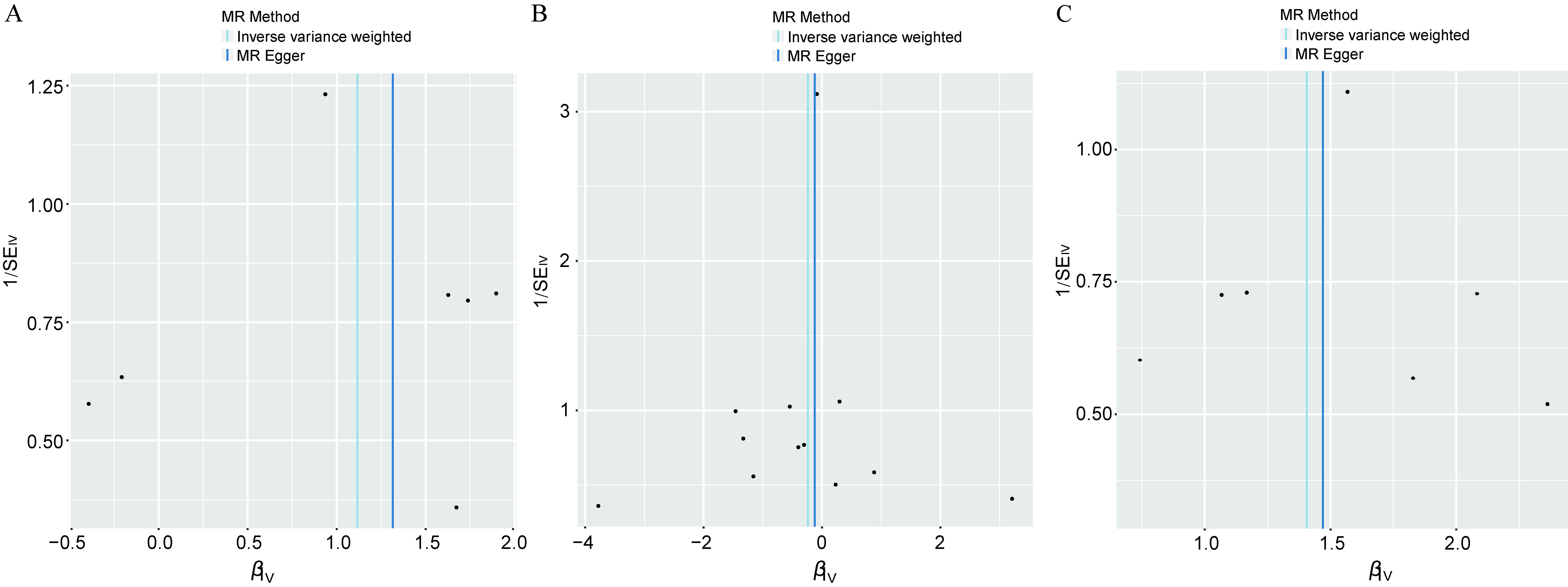


Supplementary Figure S7. Funnel plot for the effects of lipid-lowering drug targets on cystic kidney diseases.

Notes: The causal effect of individual variant against the overall estimate in the MR analysis on HMGCR−LDL−C (A), PCSK9−LDL−C (B), and NPC1L1−LDL−C (C) and cystic kidney diseases was depicted. Vertical lines denote overall estimates by the inverse variance weighted meth


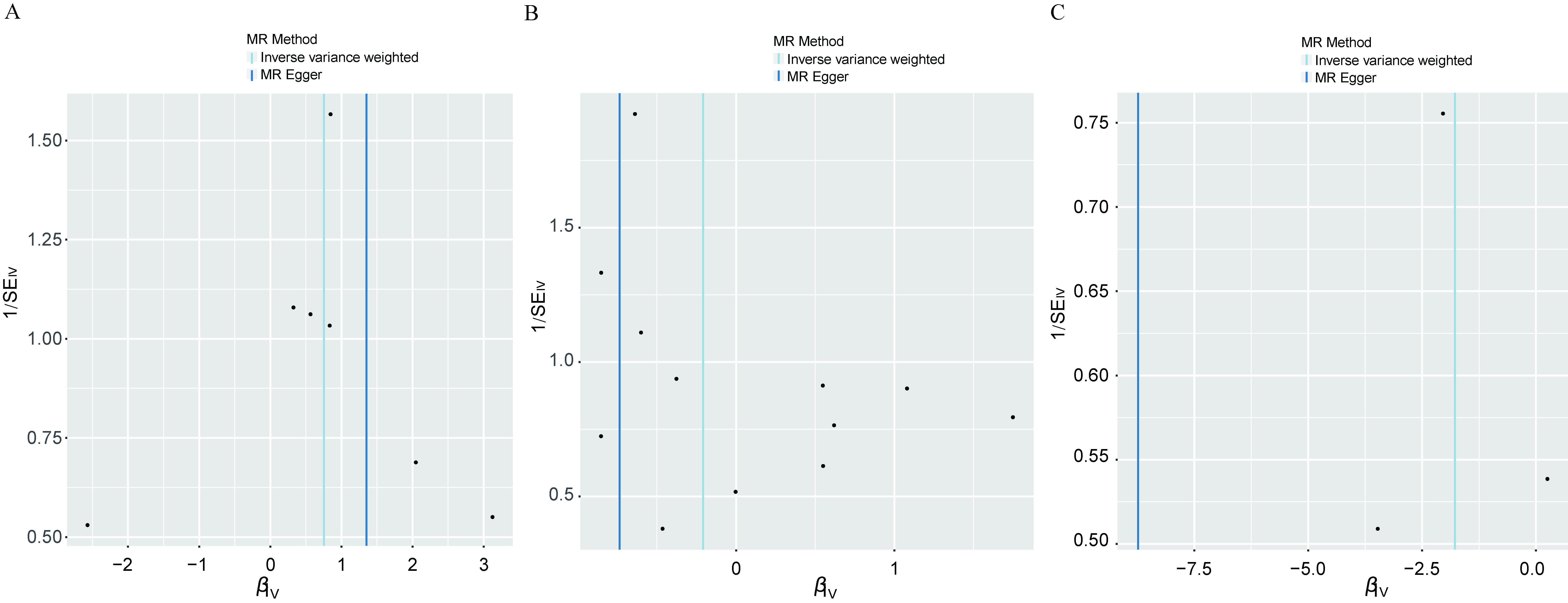


Supplementary Figure S8. Funnel plot for the effects of lipid-lowering drug targets on PKD.

Notes: The causal effect of individual variant against the overall estimate in the MR analysis on HMGCR−LDL−C (A), PCSK9−LDL−C (B), and NPC1L1−LDL−C (C) and PKD was depicted. Vertical lines denote overall estimates by the inverse variance weighted meth.

Supplementary Table S1. Basic information on lipid-lowering drugs

| Drug name | Pharmacological action | Drug targets | Gene region |
| --- | --- | --- | --- |
| Simvastatin, Atorvastatin, Rosuvastatin | Reduce LDL-C by inhibiting HMG-CoA reductase | HMGCR | GRCh37.p13: chr 5:74632993-74657941 |
| Alirocumab, Evolocumab | Reduce LDL-C by enhancing the liver’s clearance | PCSK9 | GRCh37.p13: chr 1: 55505221-555305252 |
| Ezetimibe | Reduce LDL-C reduces cholesterol absorption in the intestine | NPC1L1 | GRCh37.p13: chr 7: 44552134-44580929 |

Note: The targets HMGCR, PCSK9, and NPC1L1 were identified through a systematic search in DrugBank, a comprehensive drug and target database. The search was conducted using keywords such as "lipid-lowering agents" and "cholesterol-lowering drugs," with filters applied for drug category (e.g., lipid-modifying agents), target type (proteins/enzymes in lipid metabolism), and FDA approval status. From the results, HMGCR, PCSK9, and NPC1L1 were selected due to their central roles in lipid metabolism and their association with widely prescribed lipid-lowering drugs, such as statins, PCSK9 inhibitors, and ezetimibe.

LDL-C, low-density lipoprotein cholesterol; HMGCR, 3-hydroxy-3-methyl glutaryl coenzyme A reductase; PCSK9, proprotein convertase subtilisin-kexin type 9; NPC1L1, Niemann-Pick C1-Like 1;

Supplementary Table S2. ICD-10 code for cystic kidney disease (Q17_CYSTIC_KIDNEY_DISEA) *

| Source | Code | Name |
| --- | --- | --- |
| OUTPAT | OUTPAT_ICD10(Q612) | Polycystic kidney, adult type |
| INPAT | INPAT_ICD10(Q612) | Polycystic kidney, adult type |
| OUTPAT | OUTPAT_ICD10(Q613) | Polycystic kidney, unspecified |
| INPAT | INPAT_ICD10(Q613) | Polycystic kidney, unspecified |
| PRIM_OUT | PRIM_OUT_NOT_USED_ICD10(Q612) | Polycystic kidney, adult type. Not used in endpoint definition. POSSIBLY INACCURATE IN COMPLEX ENDPOINTS! |
| PRIM_OUT | PRIM_OUT_NOT_USED_ICD10(Q613) | Polycystic kidney, unspecified. Not used in endpoint definition. POSSIBLY INACCURATE IN COMPLEX ENDPOINTS! |
| OUTPAT | OUTPAT_ICD10(Q610) | Congenital single renal cyst |
| OUTPAT | OUTPAT_ICD10(Q6199) | Cystic kidney disease, unspecified |
| OUTPAT | OUTPAT_ICD10(Q611) | Polycystic kidney, infantile type |
| OUTPAT | OUTPAT_ICD10(Q615) | Medullary cystic kidney |
| INPAT | INPAT_ICD10(Q610) | Congenital single renal cyst |
| INPAT | INPAT_ICD10(Q611) | Polycystic kidney, infantile type |
| INPAT | INPAT_ICD10(Q6199) | Cystic kidney disease, unspecified |
| OUTPAT | OUTPAT_ICD10(Q618) | Other cystic kidney diseases |
| INPAT | INPAT_ICD10(Q615) | Medullary cystic kidney |
| PRIM_OUT | PRIM_OUT_NOT_USED_ICD10(Q61) | Cystic kidney disease. Not used in endpoint definition. POSSIBLY INACCURATE IN COMPLEX ENDPOINTS! |
| DEATH | DEATH_ICD10(Q612) | Polycystic kidney, adult type |
| PRIM_OUT | PRIM_OUT_NOT_USED_ICD10(Q6199) | Cystic kidney disease, unspecified. Not used in endpoint definition. POSSIBLY INACCURATE IN COMPLEX ENDPOINTS! |
| OUTPAT | OUTPAT_ICD10(Q6140) | Renal dysplasia of a kidney (multicystic) |
| DEATH | DEATH_ICD10(Q613) | Polycystic kidney, unspecified |
| OUTPAT | OUTPAT_ICD10(Q6141) | Renal dysplasia of both kidneys (multicystic) |
| OUTPAT | OUTPAT_ICD10(Q6148) | Nonspecified renal dysplasia |
| INPAT | INPAT_ICD10(Q618) | Other cystic kidney diseases |
| OUTPAT | OUTPAT_ICD10(Q6190) | Meckel-Gruber syndrome |
| INPAT | INPAT_ICD10(Q6148) | Nonspecified renal dysplasia |
| INPAT | INPAT_ICD10(Q6141) | Renal dysplasia of both kidneys (multicystic) |
| INPAT | INPAT_ICD10(Q6140) | Renal dysplasia of a kidney (multicystic) |
| PRIM_OUT | PRIM_OUT_NOT_USED_ICD10(Q610) | Congenital single renal cyst. Not used in endpoint definition. POSSIBLY INACCURATE IN COMPLEX ENDPOINTS! |
| PRIM_OUT | PRIM_OUT_NOT_USED_ICD10(Q611) | Polycystic kidney, infantile type. Not used in endpoint definition. POSSIBLY INACCURATE IN COMPLEX ENDPOINTS! |

* The definition of Cystic Kidney Disease in the FinnGen phenotype database is systematically established by identifying individuals with ICD-10 codes Q61.1 and Q61.9, which correspond to hospital discharge and cause of death records, respectively.

Reference: https://r8.risteys.finngen.fi/phenocode/Q17_CYSTIC_KIDNEY_DISEA#dialog-table-case-counts.
